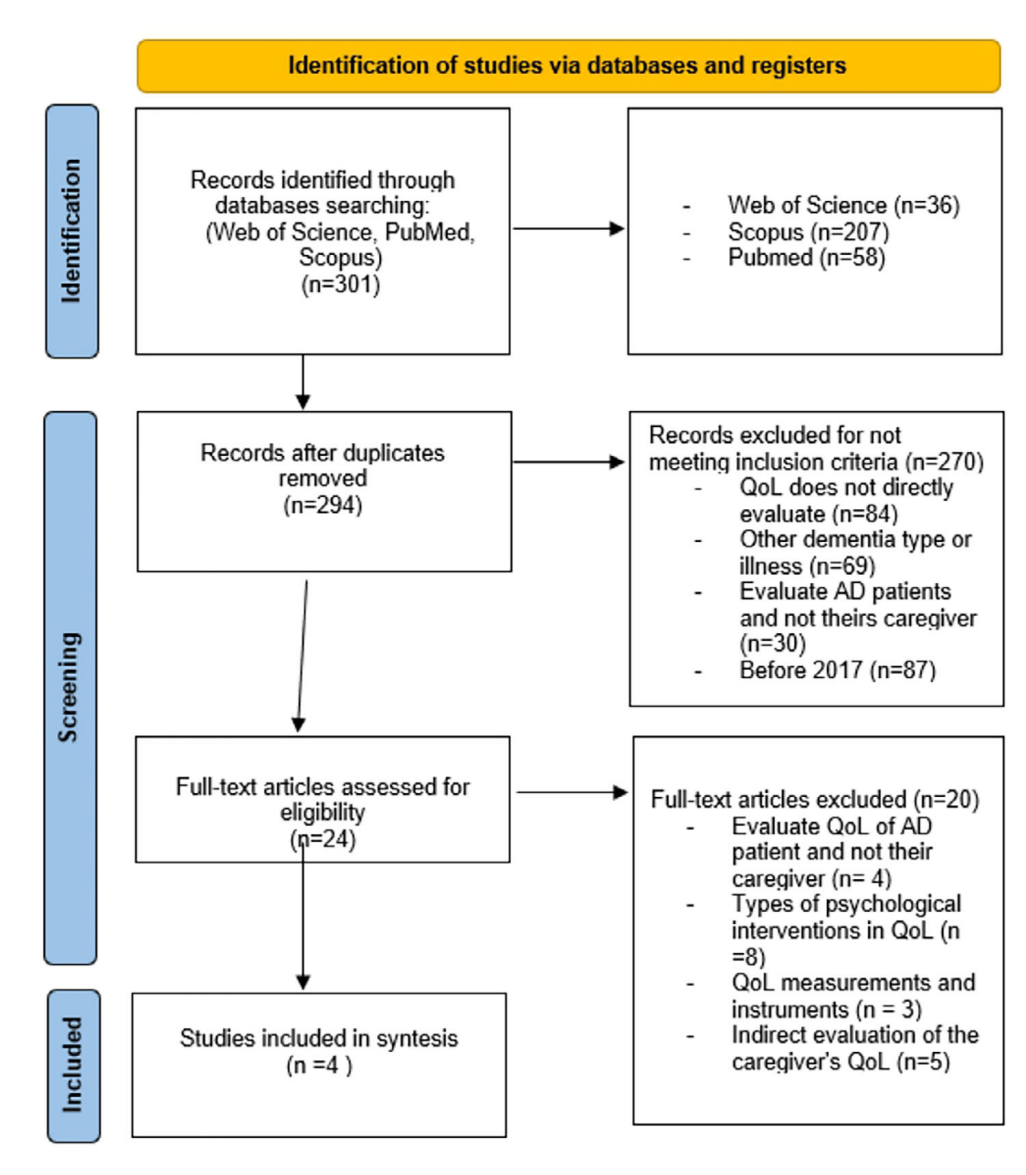# Risk factors associated with the quality of life of Alzheimer's caregivers: A systematic review

**DOI:** 10.1002/alz70858_098032

**Published:** 2025-12-24

**Authors:** Maria José Díaz Orengo, Alicia Puente Martínez, Olalla Saiz Vazquez

**Affiliations:** ^1^ University of Salamanca, Salamanca, Salamanca, Spain; ^2^ University of Burgos, Burgos, Burgos, Spain

## Abstract

**Background:**

Alzheimer Disease (AD) is a major neurocognitive disorder and leading cause of dementia. Its causes are associated with multiple biopsychosocial factors. In Chile, AD is the fourth cause of death (4.3%) and one of the main causes of disability and dependency, affecting individuals directly, caregivers, families, communities, and society. The most significant costs are associated with long‐term care, specifically adapting the living environment and providing informal caregiving, a person with an emotional bond, in most cases, who provides basic, instrumental and advanced care for the AD patient.

**Method:**

A documentary search of systematic reviews and meta‐analyses relating to the quality of life of caregivers and AD published between 2017 and 2023 was carried out in WOS, PUBMED and SCOPUS. The search strings were quality of life, AD, informal caregiver, systematic review and meta‐analysis. Primary studies in each systematic review or meta‐analysis were analyzed independently. Duplicate studies were removed. Inclusion and exclusion criteria were defined, and the PRISMA procedure, AMSTAR publication quality guidelines were applied and registered in PROSPERO.

**Result:**

Of the 301 initial studies, 19 primary studies were included (*K* = 11,483). Being a woman, low socioeconomic status, depression, anxiety, lower sense of coherence and awareness of illness were related to lower quality of life. However, positive attitudes towards care, hope, empathy, personal resources and self‐efficacy were related to better quality of life for the caregiver.

**Conclusions:**

Risk factors associated with lower caregiver quality of life include low socioeconomic status, anxiety, low sense of coherence and illness. Protective factors capable of improving quality of life include hope, empathy, personal resources and self‐efficacy. Results highlight the importance of improving the quality of life of AD caregivers.